# Acute Putrescine Supplementation with Schwann Cell Implantation Improves Sensory and Serotonergic Axon Growth and Functional Recovery in Spinal Cord Injured Rats

**DOI:** 10.1155/2015/186385

**Published:** 2015-10-13

**Authors:** J. Bryan Iorgulescu, Samik P. Patel, Jack Louro, Christian M. Andrade, Andre R. Sanchez, Damien D. Pearse

**Affiliations:** ^1^The Miami Project to Cure Paralysis, University of Miami Miller School of Medicine, Miami, FL 33136, USA; ^2^Weill Cornell Medical College, New York, NY 10021, USA; ^3^Department of Anesthesiology, Columbia University Medical Center, New York, NY 10032, USA; ^4^Department of Internal Medicine, University of South Florida Morsani College of Medicine, Tampa, FL 33612, USA; ^5^Department of Neurological Surgery, University of Miami Miller School of Medicine, Miami, FL 33136, USA; ^6^The Neuroscience Program, University of Miami Miller School of Medicine, Miami, FL 33136, USA; ^7^Interdisciplinary Stem Cell Institute, University of Miami Miller School of Medicine, Miami, FL 33136, USA

## Abstract

Schwann cell (SC) transplantation exhibits significant potential for spinal cord injury (SCI) repair and its use as a therapeutic modality has now progressed to clinical trials for subacute and chronic human SCI. Although SC implants provide a receptive environment for axonal regrowth and support functional recovery in a number of experimental SCI models, axonal regeneration is largely limited to local systems and the behavioral improvements are modest without additional combinatory approaches. In the current study we investigated whether the concurrent delivery of the polyamine putrescine, started either 30 min or 1 week after SCI, could enhance the efficacy of SCs when implanted subacutely (1 week after injury) into the contused rat spinal cord. Polyamines are ubiquitous organic cations that play an important role in the regulation of the cell cycle, cell division, cytoskeletal organization, and cell differentiation. We show that the combination of putrescine with SCs provides a significant increase in implant size, an enhancement in axonal (sensory and serotonergic) sparing and/or growth, and improved open field locomotion after SCI, as compared to SC implantation alone. These findings demonstrate that polyamine supplementation can augment the effectiveness of SCs when used as a therapeutic approach for subacute SCI repair.

## 1. Introduction

To date no treatment can completely reverse the clinical outcomes associated with spinal cord injury (SCI). Tissue injury following SCI occurs in two phases: the first involves the primary mechanical injury itself, whereas the second is progressive, mediated over the course of days to weeks after SCI by a variety of cytotoxic factors present within the injury environment. Host cellular efforts to restrict progressive tissue injury, including glial reactivity, extracellular matrix deposition, and scar formation around the lesion site, subsequently give rise to a nonpermissive environment for axon regeneration and the endogenous recovery of function [1]. This extrinsic antagonism of neurorepair is compounded by a reduction in the intrinsic growth capacity of adult central neurons [2]. Finding novel therapeutics that combat secondary injury, overcome the growth-inhibiting environment of the lesion site, and/or enhance the intrinsic capacity of neurons to regenerate is imperative for restoring function after SCI. Strategies combining cellular with pharmacological, molecular, or biomaterial approaches have shown the most promise in overcoming these obstacles to attain meaningful anatomical and functional repair [3].

**Figure 1:**
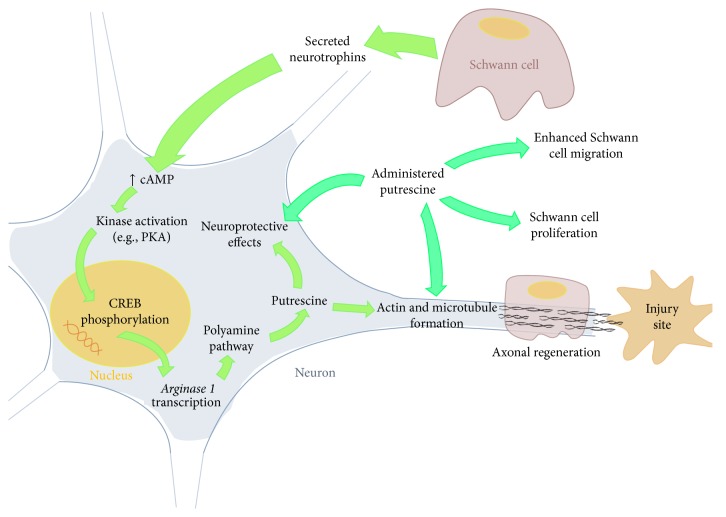
The role of putrescine in neuron and Schwann cell responses. This figure summarizes the previously reported functions of polyamines in neurons and Schwann cells within the wider literature from* in vitro* and* in vivo* studies and how these mechanisms could elicit the observed responses in the current study. Neurotrophins and growth factors secreted by implanted Schwann can elevate intrinsic cyclic AMP levels within neurons. Cyclic AMP is an important second messenger whose main effectors are downstream protein kinase signaling cascades (e.g., PKA) that result in the phosphorylation and activation of the constitutive transcription factor CREB. Bound to the nuclear DNA, CREB is known to enhance the expression of a number of genes that include* arginase 1*, which encodes the first enzyme of the polyamine pathway to generate endogenous putrescine. Putrescine produces a number of neuroprotective effects by scavenging free radicals and suppressing excitotoxicity. Putrescine also promotes the reorganization of actin and microtubules, allowing axons to grow. Administered putrescine is known to be taken up by both the neuron, bolstering the effects of the endogenous polyamines, and SCs, encouraging their migration and proliferation* in vitro*. We hypothesize that SC implants and administered putrescine work in concert to augment each other's ability to promote neuropreservation and neuroregeneration following SCI.

One encouraging adjunctive strategy for both surmounting the growth-inhibitory environment of the lesion and stimulating intrinsic regeneration has been the elevation of cyclic adenosine monophosphate (cyclic AMP), a second messenger molecule [4]. Sustained elevation of cyclic AMP has been shown to facilitate the extension of neurites across inhibitory substrates such as myelin and myelin-associated glycoprotein, to promote neuroprotection by elevating antiapoptotic proteins, and to suppress immune cell activation and ensuing inflammation [4]. The primary target of the cyclic AMP pathway is the DNA-bound, constitutive transcription factor CREB, which upon phosphorylation by the cyclic AMP-activated protein kinase A (PKA) initiates the expression of numerous genes, including those that encode certain neuropeptides, neurotrophins, and arginase 1 (a crucial enzyme in the synthesis of polyamines; Figure 1) [5]. Downstream products of arginase 1, including the polyamine putrescine, have been shown to mediate several of cyclic AMP's effects on neurite outgrowth [6]. Supplementing glial-derived neurotrophic factor gene delivery with the substrate of arginase 1, L-arginine, demonstrated significant reductions in contusion size in traumatic brain injury models, although without improvement in cognitive function [7]. In paradigms of SCI, augmenting peripheral nerve grafts with acidic fibroblast growth factor supplementation increased* arginase 1* expression and polyamine synthesis and was associated with favorable M2 macrophage responses and axonal regeneration [8, 9]. Additionally, human mesenchymal stromal cell implants that overexpressed a key enzyme in the synthesis of polyamines, arginine decarboxylase, resulted in improved locomotor recovery and reduced scar formation following SCI [10]. Based upon our previous studies demonstrating the ability of cyclic AMP elevation to improve the effectiveness of SC implants after SCI and the growing evidence that polyamine synthesis may mediate several of cyclic AMP's downstream effects on axon regeneration [3, 4, 6], we investigated whether the supplementation of SC implants with putrescine, given either within 30 minutes or following a 1-week delay after contusive SCI, could improve SC implant-host integration, axon growth support, and locomotor recovery in an experimental contusive SCI paradigm.

## 2. Materials and Methods

### 2.1. Schwann Cells

SCs were obtained from the sciatic nerves of adult female Fischer rats (Harlan Co., Indianapolis, IN) as previously described [11, 12]. SCs were grown to confluence and thrice passaged onto new dishes. Following previous methods, SC purity for implantation was measured to be between 95% and 98%, according to p75 immunoreactivity [13].

### 2.2. Animals

Adult female Fischer rats (Harlan Co., *N* = 34; 180–200 g) were housed according to the guidelines recommended by the NIH and the Guide for the Care and Use of Animals. All animal procedures were approved by the Institutional Animal Care and Use Committee of the University of Miami. Prior to surgery, rats were anesthetized (70 mg/kg ketamine, 5 mg/kg xylazine) by intraperitoneal injection.

### 2.3. Moderate Thoracic Contusion Injury

The MASCIS weight drop device was used to induce a moderate thoracic (T9) contusion injury [14]. A laminectomy was performed at the T8 thoracic vertebra prior to injury in order to expose the dorsal surface of the underlying spinal cord (T9) without perturbing the dura mater. The exposed spinal cord was then subjected to a moderate injury by dropping a 10 g rod from a height of 12.5 mm. Contusion impact height, velocity, and compression distance were monitored for all animals. Those which had height or velocity errors exceeding 6% or a compression distance that was not within a range of 1.25–1.75 mm were immediately excluded [3, 15]. According to these injury parameter criteria no animals required exclusion from the study. Following injury, the muscles were sutured in layers and the skin was closed with metal wound clips. Animals were allowed to recover in warmed cages with easy access to food and water. Gentamicin (5 mg/kg; Abbott Laboratories, North Chicago, IL) was intramuscularly administered immediately after the surgery and repeated daily for 7 days. The analgesic buprenorphine (0.03 mg/kg; Reckitt Benckiser, Richmond, VA) was delivered subcutaneously following surgery and then daily for 2 days, accompanied by postoperative treatment as described previously [16].

### 2.4. Subcutaneous Minipump Delivery of Putrescine

**Figure 2:**
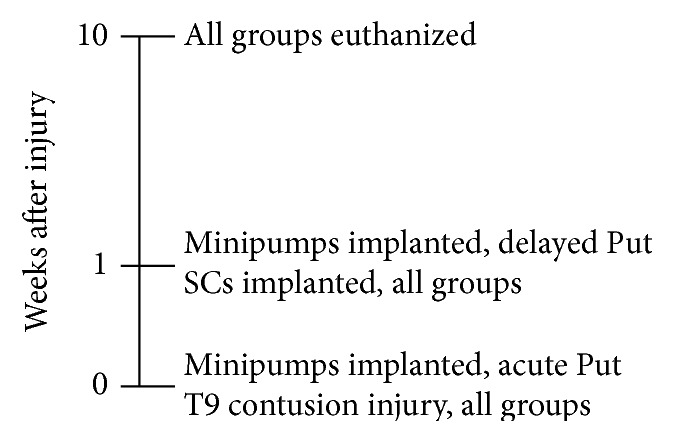
Methodology timeline: all groups received a T9 contusion injury at the 0-week time point. The Acute Put + SC group also received the putrescine-administering minipumps within 30 minutes of injury. At 1 week after injury all groups received SC. Also at 1 week after injury the Delayed Put + SC group received the putrescine-administering minipumps. All groups were euthanized and prepared for tissue processing at 10 weeks after injury.

Prior to SCI induction, animals were randomly assigned into one of the following three treatment groups: (i) SC implant with 0.9% physiological saline (vehicle) control (*n* = 11), (ii) SC implant with acute putrescine (administered within 30 minutes of injury, *n* = 14), and (iii) SC implant with delayed putrescine (administered 1 week after injury at time of implantation, *n* = 9). Putrescine (100 mM) was delivered at a rate of 0.5 *μ*L/hr using two sequentially implanted subcutaneous Alzet minipumps (model 2001; Durect Corp., Cupertino, CA) for a 2-week administration period; emptied pumps were replaced after 1 week [17].  Figure 2 presents a timeline of the methodology.

### 2.5. Schwann Cell Implantation

Before implantation, SCs were trypsinized, harvested, counted, resuspended in DMEM/F12 media as aliquots and placed on ice prior to surgery. One week after injury the rats were anesthetized with 2% halothane and the injured spinal cord was reexposed. Rats then received fluid implants of SCs as described elsewhere [3]. Cells were implanted within 2 hours of preparation. For implantation, all animals received 2 × 10^6^ SCs in 6 *μ*L of DMEM-F12 media into the epicenter of the contused area, injected at the midline to a depth of 1 mm. Injections were performed at a rate of 2 *μ*L/min using a 10 *μ*L siliconized Hamilton syringe with a pulled, beveled glass pipette tip (120 *μ*m diameter), which was held in a micromanipulator with an attached microinjector (World Precision Instruments, Sarasota, FL). The injection pipette was held in place for an additional 3 min postinjection to minimize leakage upon withdrawal. One animal was excluded from the study due to a malfunction of the microinjector during cell transplantation. Following the injection, the muscle layers and the skin were closed separately and animals received postoperative care as described above.

### 2.6. Histology

Rats were euthanized at 10 weeks after injury (9 weeks after implantation; 100 mg/kg ketamine, 10 mg/kg xylazine) and transcardially perfused with 4% paraformaldehyde (PFA, 0.1 M, pH 7.4). The tissue was then dissected and sectioned with a freezing microtome for immunohistochemical analysis according to previous procedures [3]. Spinal cords (2 cm) encompassing the injury epicenter were sectioned sagittally at 40 *μ*m into five series free-floating.

### 2.7. Immunohistochemistry

As previously described, every fifth sagittal section was immunochemically stained for subsequent fluorescent microscopic analysis using a double labeling procedure involving monoclonal and polyclonal antibodies [15]. These antibodies were raised against the low-affinity neurotrophin receptor (p75^NTR^; 1 : 5,000; Abcam, Cambridge, MA), serotonin (5HT; 1 : 5,000; Immunostar, Hudson, WI), or calcitonin gene related peptide (CGRP; 1 : 1,000; Peninsula Laboratories, San Carlos, CA) for identifying the implanted SCs or delineating the growth of specific descending or ascending axonal populations, respectively, within the p75^+^ SC implants. Sections were then washed and incubated with corresponding fluorescent secondary antibodies (Alexa 488- or Alexa 594-conjugated goat anti-rabbit or anti-mouse antibodies, 1 : 200; Molecular Probes, Eugene, OR). Sections were mounted onto Snowcoat X-tra slides (Surgipath, Richmond, IL) and cover-slipped with Vectashield mounting medium (Vector Laboratories, Burlingame, CA) containing the nuclear dye Hoechst for storage at 4°C.

### 2.8. Stereological Quantification of Implant Area

In one series of sections, the average area of p75-positive cellular immunoreactivity was quantified across three sections containing the center of the SC implant. Expression of p75 permits delineation of the implant and identification of the implant-host cord interface. For this analysis, stained sections were visualized and quantitatively assessed using an unbiased method employing computer-assisted fluorescence microscopy and Neurolucida software (version 4.5; MicroBrightField Bioscience, Williston, VT). The p75^+^ area for each of the three sections that comprised the center of the SC implant was traced under a 20x objective, quantified using Neurolucida, and then the SC implant area calculated for each animal as the average p75^+^ area across the three sections [18].

### 2.9. Determination of 5HT and CGRP Fiber Growth

Quantification of 5-hydroxytryptophan (5HT) labeled axon growth along the rostral-caudal axis of the spinal cord at distances of 1000, 500, 100, and 0 *μ*m rostral to the center of the implant (as identified using p75 immunoreactivity) was performed on the second series of sections using 63x objective under oil immersion. Calcitonin gene related peptide (CGRP) labeled axons were quantified similarly, but at 1000, 500, 100, and 0 *μ*m caudal to the center of the implant. The average number of fibers per section was obtained by counting those immunostained axons that crossed imaginary lines perpendicular to the rostral-caudal axis at the 1000, 500, 100, and 0 *μ*m intervals from the center of the implant [19]. The total number of axons counted for a given animal was summated across the sections analyzed (~10–12 sections per animal) and then divided by the number of sections to determine the number of fibers per section (f/s) at each distance [4].

### 2.10. Behavioral Testing

The open field BBB locomotor test developed by Basso et al. was employed to assess weekly gross locomotor performance after SCI for 10 weeks [20]. In addition, a subscore analysis of the BBB that allows for evaluation of hind limb positioning and placement as well as balance and tail use on a 0–13 point scale was also used as described previously by our group [3]. Lastly, deficits in descending motor control were examined by counting footfall errors on an irregularly spaced horizontal grid walk at the end of the study, prior to animal perfusion [4]. All behavioral tests were conducted by two individuals blinded to the animal's treatment.

### 2.11. Statistical Analysis

A one-way ANOVA and subsequent Bonferroni post hoc test were used to compare counts of immunostained axons among groups. For analysis of weekly functional recovery patterns following implantation (BBB and BBB subscore), a mixed factorial (repeated measures) ANOVA followed by the Tukey-Kramer posttest was employed. Differences were acknowledged to be statistically significant at *p* < 0.05 ^*∗*,#^, <0.01^*∗∗*,##^, and <0.001^*∗∗∗*,###^, compared to the SC only control or the other temporal treatment group, as indicated. All errors are given as standard errors of the mean.

## 3. Results

### 3.1. SC Implant Size Increased with Delayed Putrescine Supplementation

**Figure 3:**
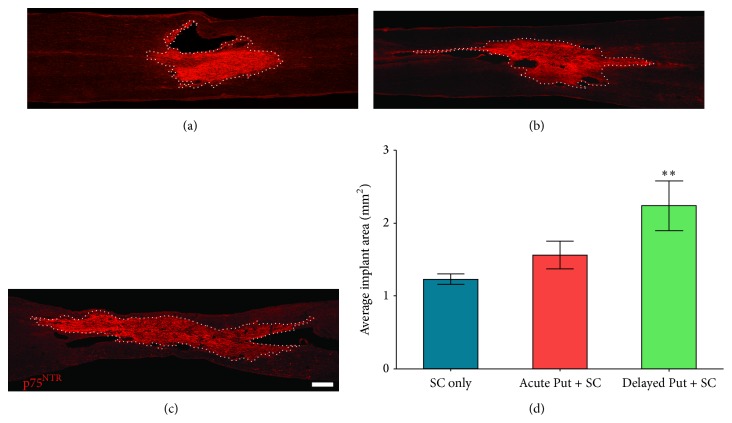
Delayed putrescine supplementation significantly increases SC implant size. To determine the effect of putrescine on the size of the implant, immunostaining for p75^NTR^ was performed and the area of immunoreactivity quantified. Images from representative animals show the extent of p75^NTR^ immunoreactivity among the different SC implant groups: (a) vehicle control, (b) immediate putrescine, and (c) delayed putrescine. Scale bar = 500 *μ*m. Dotted white lines indicate the extent of the SC implant. Stereological quantification of the implant area shows an increase in size when putrescine is administered in a delayed fashion, but not acutely, as compared to the SC with vehicle control (d). Statistical significance indicated at ^*∗∗*^ *p* < 0.01 compared to SCs with vehicle control group.

Statistical comparison of SC implant size among control and treatment cohorts showed a significant effect of treatment (*F*_2,31_ = 5.784, *p* < 0.05). Compared to the implant size in the SC only group (1.23 ± 0.07 mm^2^), those animals receiving putrescine administration beginning at the time of implantation exhibited 1.8-fold increase in implant area (2.24 ± 0.34 mm^2^; *t*_33_ = 3.391, *p* < 0.01; Figures 3(a), 3(c), and 3(d)) as identified using p75 immunochemistry (a marker highly expressed on SCs) [13]. In contrast, putrescine administration beginning at the time of injury was without effect (1.56 ± 0.19 mm^2^, *p* < 0.05).

### 3.2. Putrescine Improves Serotonergic Fiber Growth into the SC Implant

**Figure 4:**
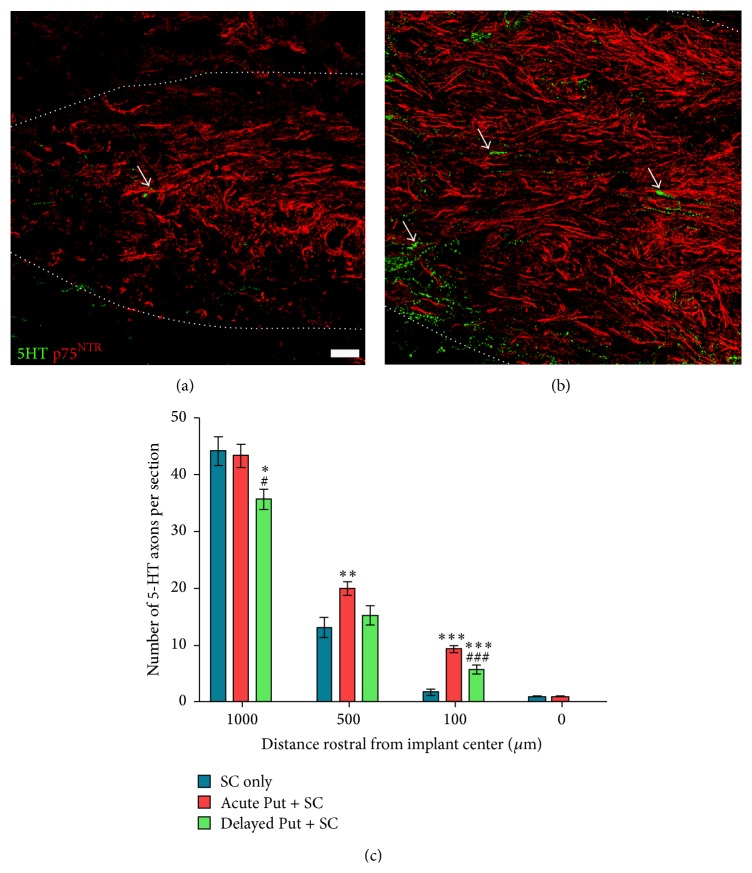
Putrescine supplementation enhances serotonergic axon numbers within the SC implant. Immunochemical staining for serotonergic axons (green, representative examples indicated by white arrows) shows many more axons within the rostral part of the SC implant (identified by p75 labeling, red) following (b) acute putrescine delivery compared to (a) when vehicle is given. Scale bar = 20 *μ*m. Dotted white lines indicate the extent of the SC implant. (c) Stereological analysis shows a significant increase in 5HT^+^ axon numbers with putrescine; the greatest effects are seen further from the center of the SC implant (i.e., closer to rostral host cord SC implant interface) and with acute administration. Statistical significance indicated at ^*∗*^ *p* < 0.05, ^*∗∗*^ *p* < 0.01, and ^*∗∗∗*^ *p* < 0.001 compared to the vehicle control group and ^#^ *p* < 0.05 and ^###^ *p* < 0.001 compared to acute putrescine treatment.

Serotonergic axons descending from the reticular formation and raphe nuclei of the brainstem were identified penetrating the SC implant using immunostaining for 5HT (Figures 4(a)–4(c); white arrows). For all animal groups there were few serotonergic axons that were able to penetrate beyond the center of the SC implant and no treatment effect was observed at this location (*F*_2,31_ = 1.414, *p* > 0.05; Figure 3(c)). However, statistical comparison of 5HT axon numbers within the rostral part of the SC implant showed a significant treatment effect (*F*_2,31_ = 4.104, *p* < 0.05 at 1000 *μ*m, *F*_2,31_ = 6.200, *p* < 0.01 at 500 *μ*m, and *F*_2,31_ = 41.15, *p* < 0.001 at 100 *μ*m from the center of the implant). Acute putrescine supplementation significantly increased the number of serotonergic fibers within the proximal SC implant compared to the vehicle control: an average of 20 ± 1.2 5HT^+^ fibers per section (f/s) at 500 *μ*m (*t*_33_ = 4.829, *p* < 0.01) and 9 ± 0.6 f/s at 100 *μ*m (*t*_33_ = 12.82, *p* < 0.001) was quantified rostral to the center of the SC implant, while in vehicle controls 13 ± 1.8 5HT^+^ f/s and 2 ± 0.4 f/s, respectively, were observed at these distances (Figures 4(a)-4(b)). In addition, many more 5HT^+^ axons were observed crossing the host-SC implant interface in acute putrescine treated animals (Figures 4(a)-4(b); white arrows). Delayed putrescine supplementation produced a significant increase in 5HT^+^ axon numbers only at 100 *μ*m rostral to the center of the SC implant (5.6 ± 0.8 f/s, *t*_33_ = 6.011, *p* < 0.001).

### 3.3. Putrescine Supplementation Improves Sensory Fiber Growth into the SC Implant

**Figure 5:**
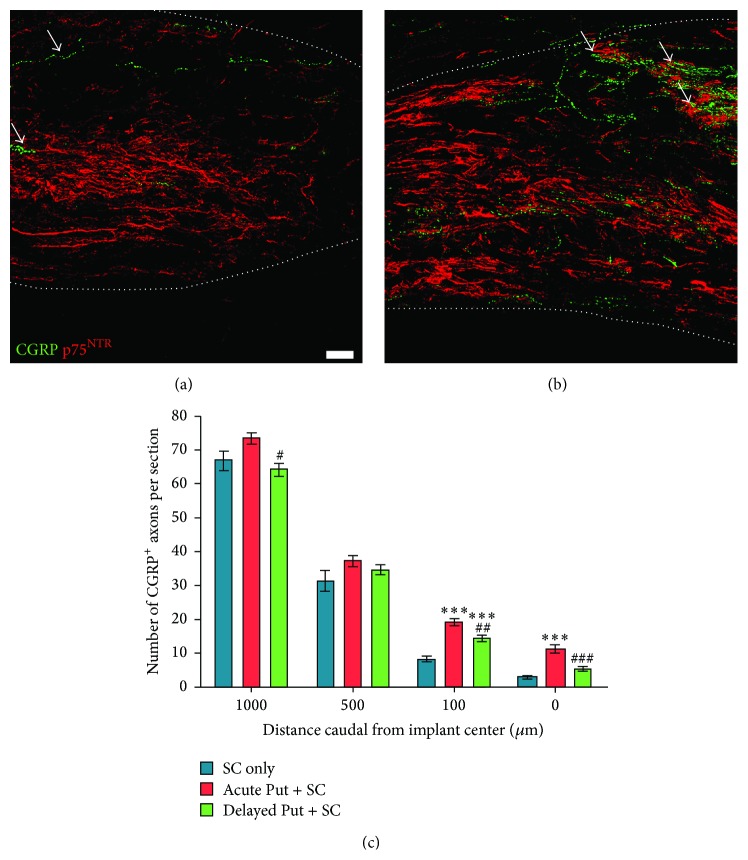
Putrescine administration increases peptidergic axon numbers within the SC implant. Immunochemical staining for peptidergic axons with CGRP (green, representative examples indicated by white arrows) shows that many more axons are present within SC implants (p75 labeling, red) following (b) acute putrescine delivery compared to (a) when vehicle is given. Scale bar = 20 *μ*m. Dotted white lines indicate the extent of the SC implant. (c) Stereological analysis revealed a significant increase in CGRP^+^ axon numbers with putrescine: the greatest effects are observed within the SC implants with acute putrescine administration. Statistical significance indicated at ^*∗∗∗*^ *p* < 0.001 compared to the vehicle control group and ^#^ *p* < 0.05, ^##^ *p* < 0.01, and ^###^ *p* < 0.001 compared to acute putrescine treatment.

Small diameter sensory axons originating from dorsal root ganglia that were immunoreactive for CGRP were quantified within the SC implant (Figures 5(a)–5(c); white arrows). Statistical comparison of CGRP^+^ axon numbers among groups showed a significant treatment effect at both 100 *μ*m caudal to (*F*_2,31_ = 35.17, *p* < 0.001) and within the center of the SC implant (*F*_2,31_ = 16.16, *p* < 0.001). At 100 *μ*m caudal to the center of the SC implant, acute and delayed putrescine supplementation resulted in 2.3-fold (19 ± 1.0 f/s, *t*_33_ = 11.86, *p* < 0.001) and 1.7-fold (14 ± 0.8 f/s, *t*_33_ = 5.941, *p* < 0.001) increases in CGRP^+^ axon numbers, respectively, compared to the vehicle control (8.3 ± 0.77 f/s). In addition, acute putrescine administration (11 ± 1.2 f/s) significantly increased CGRP^+^ axon ingrowth within the center of the SC implant compared to the vehicle control (2.8 ± 0.4 f/s, *t*_33_ = 9.491, *p* < 0.001, a 3.9-fold increase). Acute putrescine was also significantly more effective in promoting CGRP^+^ axon growth than delayed delivery at both 100 *μ*m caudal to (14 ± 0.8 f/s, *t*_33_ = 4.935, *p* < 0.01, a 1.4-fold increase) and within the center of the SC implant (5.3 ± 0.7 f/s, *t*_33_ = 6.257, *p* < 0.001, a 2.1-fold increase).

### 3.4. Putrescine Supplementation Improves Locomotor Recovery

**Figure 6:**
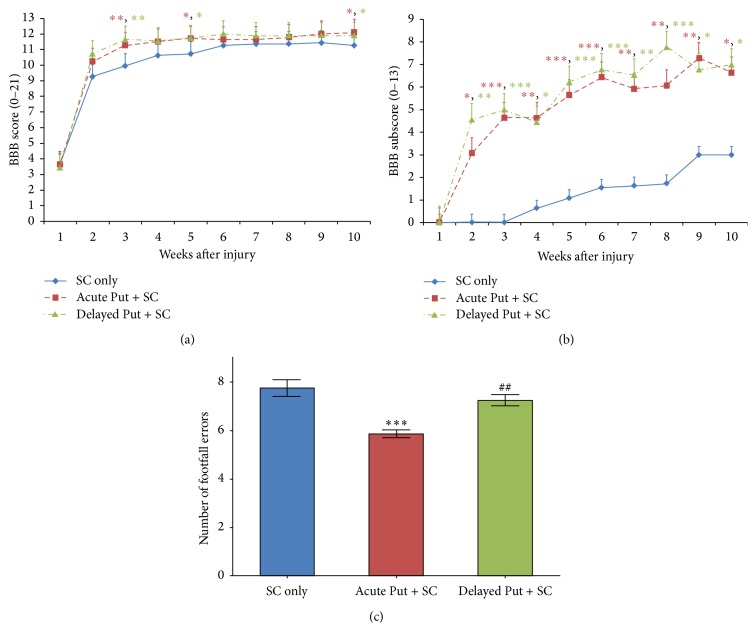
Putrescine supplementation enhances the functional outcomes obtained with SC implants. Assessment of open field locomotor performance with the (a) BBB score and subsequent (b) BBB subscore analysis shows that animals receiving either acute or delayed putrescine have improved walking behavior over SC implanted, vehicle controls. Improvements in foot and tail positioning, as measured by the BBB subscore, exhibit a more acute and stable pattern of recovery. (c) Recording the number of footfalls that occur while traversing a horizontal grid walk test also showed an improvement in hind paw positioning when acute putrescine was administered (red) compared to vehicle only controls (blue). Delayed putrescine (green) was without effect. Statistical significance indicated at ^*∗*^ *p* < 0.05, ^*∗∗*^ *p* < 0.01, and ^*∗∗∗*^ *p* < 0.001 compared to the vehicle control group or ^##^ *p* < 0.01 compared to acute putrescine.

Locomotor performance and functional recovery following SCI, SC implantation, and putrescine treatment were assessed using the BBB score, BBB subscore, and the irregularly spaced grid walk test. Statistical comparison of BBB scores among groups showed a significant treatment effect both at endpoint (*F*_2,31_ = 4.303, *p* < 0.05) and at various time points after implantation (*F*_2,31_ = 8.520, *p* < 0.01 at week 3; *F*_2,31_ = 4.771, *p* < 0.05 at week 5; Figure 6(a)). Compared to the SC implant, vehicle controls (11.3 ± 0.3, 10 weeks after SCI), open field locomotor performance was significantly greater at endpoint in both the acute (12.1 ± 0.1, *t*_33_ = 4.079, *p* < 0.05) and delayed (11.9 ± 0.3, *t*_33_ = 3.929, *p* < 0.05) putrescine with SC implant treatment groups. Statistical comparison of BBB subscores among groups showed a significant effect of treatment at all weeks after implantation (*F*_2,31_ = 4.320, *p* < 0.05, 10 weeks after SCI; Figure 6(b)). Compared to SC implant, vehicle controls (3.0 ± 0.3, 10 weeks post-SCI), hind paw placement and tail positioning were significantly superior in both acute (6.6 ± 1.1, *t*_33_ = 3.631, *p* < 0.05) and delayed (7.0 ± 1.4, *t*_33_ = 3.574, *p* < 0.05) putrescine with SC implant treatment groups. Statistical comparison of the number of footfall errors on the grid walk among groups showed a significant effect of treatment (*F*_2,31_ = 16.76, *p* < 0.001; Figure 6(c)). Acute putrescine administration with SC implantation resulted in significantly better hind paw placement performance as evidenced by a lower number of footfalls on the grid walk (5.9 ± 0.2 footfalls) compared to both delayed administration (7.3 ± 0.2 footfalls, *t*_33_ = 3.832, *p* < 0.01) and the SC implant, vehicle controls (7.8 ± 0.4 footfalls, *t*_33_ = 5.538, *p* < 0.001).

## 4. Discussion

Bridging the hostile environment of the injured spinal cord with implanted SCs has been shown to be an effective foundation approach for a number of combinatorial repair strategies in a diverse array of experimental SCI models [3, 4, 19]. In addition to ensheathing and myelinating axons, SCs provide growth-promoting neurotrophins, which may limit tissue loss and encourage axons to overcome the growth-inhibitory injury milieu. Importantly, SCs can be safely harvested from a patient's own peripheral nerve and autologously implanted back into his spinal cord, a major advantage to their use in the clinical setting [4]. However, SC implants by themselves cannot entice significant numbers of axons to exit the injury site, are unable to support robust growth of most supraspinal axon populations except under specific conditions, and provide only modest improvements in functional outcome [13, 15, 21]. In order to mediate significant anatomical repair and/or functional improvements, SC implants must be augmented with various pharmacological, molecular, or biomaterial approaches that overcome intrinsic or extrinsic inhibitors of axon growth, such as neurotrophin supplementation, chondroitinase ABC, polysialic acid, matrix suspension, or cyclic AMP elevation [4, 19, 22].

In the current study we have demonstrated that the administration of the initial polyamine, putrescine, when combined with SC implants, significantly improved the size of the implant ~2-fold and thus potentially the number of surviving, implanted SCs. In addition, acute putrescine administration enhanced the sprouting and/or sparing of both descending serotonergic and ascending peptidergic axons within the implant/lesion site by up to 400%. Delaying the administration of putrescine mirrored the effects of the acute model, but generally with a reduced effectiveness. The improvements in implant size and axon growth support following putrescine administration were accompanied by enhanced functional recovery, including significant gains in open field locomotor ability and hind paw placement compared to animals receiving SC implants alone.

We have previously shown the therapeutic potential of combining SC implantation with cyclic AMP elevation [4]. Elevated levels of cyclic AMP are critical in facilitating the growth of axons over inhibitory substrates, such as myelin* in vitro *[2], and across the growth-retarding milieu of the injured spinal cord [3]. The upregulation of* arginase 1*, a downstream effector of cyclic AMP that synthesizes the precursor for polyamines, has similarly been shown to enhance axonal regeneration, whereas blocking ornithine decarboxylase, the rate limiting enzyme which decarboxylates the product of arginase 1 into putrescine, attenuates the ability of dibutyryl cyclic AMP and brain-derived neurotrophic factor to overcome growth antagonism by myelin-associated inhibitors [6–10]. The polyamine putrescine can further be converted into the polyamines spermidine and spermine; however the reverse reactions release toxic aldehyde byproducts, making putrescine the polyamine of choice for experimental or clinical application of polyamines [23, 24]. In addition to its axon growth-promoting abilities, putrescine has also been shown to act as a neuroprotectant, preventing neuronal cell death following ischemia or trauma, likely through its antioxidant and free radical scavenging actions [25, 26]. In addition, polyamines are thought to be incorporated into the elF5A factor, which has been reported to mediate the neurotrophic and neuroprotective effects of nerve growth factor [27]. Taken together, these studies evidence the therapeutic benefit of using polyamines to enhance the functionality of cellular implants after SCI.

In the current study, SC implants were visualized immunohistochemically using an antibody against p75, the low-affinity nerve growth factor receptor highly expressed in SCs both in culture and following spinal cord implantation. The p75 receptor has been used previously to identify SC implants* in vivo* [4]. We show that delayed, but not acute, supplementation of SC implants with putrescine resulted in an 80% increase in the size of the implant. From the overlap in administration time between the acute and delayed paradigms and the absence of an effect with acute putrescine, it is unlikely that the observed enhancement in implant size is due to a direct stimulation of SC survival or proliferation by putrescine but instead requires the presence of additional host-supplied factors. As both endogenous SC in-migration and axonal ingrowth occur with a 1–3-week delay after SCI, the later delivery of putrescine may enhance endogenous SC in-migration and/or work in concert with signals from in-growing axons to stimulate proliferation of implanted SCs [3, 28]. Following traumatic SCI, endogenous SCs have been shown to dedifferentiate in the spinal nerve roots, migrate into the injury site, proliferate, and significantly upregulate p75 expression [28, 29]. Migration and proliferation of endogenous SCs occur between 1 and 3 weeks following injury, which temporally corresponds with our delayed putrescine supplementation paradigm. In addition, axon presence is one of the strongest signals for inducing SC mitogenesis and proliferation, and there may be insufficient growth of axons within the implant/lesion site during the first week after SCI to stimulate SC proliferation acutely. Therefore, implanted SC proliferation and an ensuing increase in implant size may be best benefited by the combination of putrescine and axonal growth signals during the delayed administration period. Whether an increased area of p75 immunoreactivity denotes an enhanced proliferation of implanted SCs or an enhanced proliferation and/or migration of endogenous SCs into the lesion is difficult to determine unequivocally without cell tracking methods. However, depletion of polyamines in a variety of cell types has been shown to impair cell migration by reorganizing the F-actin of lamellipodia, impeding microtubule reformation, and preventing the accumulation of *β*-actin and *α*-tubulin mRNA [30–32]. Thus putrescine could enhance the migratory ability of endogenous SCs. On the other hand, SCs are largely mitotically quiescent under normal physiological conditions, proliferating only during development, Wallerian degeneration, or a demyelinating condition or insult. The enhanced proliferative capacity of SCs during Wallerian degeneration has been closely associated with an elevation of ornithine decarboxylase activity, suggesting that putrescine supplementation may promote the proliferation of either endogenous or exogenous SCs within the lesion [33, 34]. Amplified activity of ornithine decarboxylase and polyamines and the elF5A translation factor derived from polyamines have all been shown to upregulate proliferation of a number of cell types [35, 36]. Further investigations are needed to ascertain the exact mechanism by which p75 and SC implant size are enhanced following SCI, implantation, and delayed supplementation of putrescine.

In addition to mediating cell migration and proliferation, putrescine and other downstream polyamines have also been shown to promote cell survival. Deficiencies in putrescine levels cause swelling of the endoplasmic reticulum and Golgi bodies, as well as the disappearance of stress fibers, all of which are hallmarks associated with necrosis [37]. Intracellular polyamines can alter ion transport, either blocking K^+^ channels to prevent neuron excitotoxicity or promoting Ca^2+^ influxes [38, 39]. Polyamine-induced ion influxes have been shown to be required to induce the release of 5HT synaptosomes, which may help restore serotonergic axon functionality after SCI [40]. SC implants, when used in combinatory approaches, were previously shown to improve the regeneration of serotonergic fibers following SCI [4]. Here we observed that supplementing SC implants with putrescine significantly increased the number of serotonergic fibers proximal to the center of the implant/lesion, an effect strengthened by administering the putrescine acutely. Serotonergic axon projections within the spinal cord are believed to be important for the recovery of locomotion following injury [41]. While it is difficult to determine whether the increase in the numbers of serotonergic fibers is due to the regeneration of axotomized axons, collateral sprouting from existing serotonergic fibers, or sparing of axons, an enhanced presence of serotonergic axons following putrescine administration may provide important stimulatory effects for improved locomotor functioning [42]. Future use of a complete spinal cord transection paradigm with SC grafting [12] would provide a means for identifying whether such 5HT axon growth is due to regeneration, sprouting, or sparing and, when combined with neuroanatomical tracing, whether the serotonergic axons are from supraspinal or intraspinal neurons.

The growth of primary sensory afferents, immunoreactive for CGRP, which have terminations in the superficial lamina of the spinal cord and are associated with transmission of pain, also benefitted from the delivery of putrescine, with enhanced axon numbers within the center of the SC implant. CGRP-positive neurons have also been implicated in the modulation of fine motor control and the control of posture and thus their growth could be indicative of changes in locomotor function [43]. Primary dorsal horn afferent fibers are known to sprout into more medial lamina of the spinal cord following SCI, again making it challenging to ascertain whether the increasing number of CGRP-positive fibers was due to regenerating sensory afferents or collateral sprouts [44]. Like 5HT^+^ axon growth, acute administration of putrescine following SCI and SC implantation generated the greatest amount of CGRP^+^ fiber growth, almost four times as many fibers compared to SCs alone and more than twice as many fibers as delayed supplementation. These CGRP^+^ axons may have arisen from DRGNs present at the level of the lesion or from DRGNs located more distally. Further investigation is needed to explore the mechanisms by which putrescine encourages primary sensory afferents to surmount the inhibitory environment of the host cord-implant interface and penetrate deeply into the SC implants as well as the use of tracing techniques to identify the level(s) from which responding DRGNs are located.

Putrescine supplementation of SC implants also resulted in significant functional improvement, as measured in the open field with the BBB score and on the grid walk test. Irrespective of the timing of putrescine delivery, enhanced locomotor ability, as measured by using the BBB score and subscore, was obtained with treatment compared to SC implanted, vehicle controls. Overall, both acute and delayed putrescine administration generated similar improvements in hind limb performance and tail posturing, including a one-point higher BBB score and five-point higher BBB subscore. Only the acute delivery of putrescine, however, reduced footfall errors on the horizontal grid walk test, by 25% compared to the vehicle control. The effectiveness of acute putrescine only on this outcome measure would indicate that tissue preservation would be a likely mechanistic explanation for this effect. The observed functional improvements following supplementation of SC implants with putrescine are in addition to those that are known to be provided when SC implants alone are used after SCI [4, 13]. The most pronounced behavioral improvement was displayed by the BBB subscore, in which a persistent improvement in outcome was observed starting at 1 week after implantation. This highly acute benefit may imply that these effects acted through putrescine-mediated neuro- or axopreservation. The later improvements in BBB score and the continuous improvement in subscore may in turn be due to the reparative actions instigated by the initial delivery of putrescine.

## 5. Conclusions

In conclusion, we supply evidence that modulating polyamines in conjunction with cell implantation is an effective approach for enhancing the functionality of the implant as well as locomotor recovery after SCI. Future research should seek to gain a greater understanding of the mechanisms underlying putrescine's effects on SC function, migration, and survival, as well as its ability to promote axon sparing, growth, and/or sprouting.

## References

[1] Yiu G., He Z. (2006). Glial inhibition of CNS axon regeneration. *Nature Reviews Neuroscience*.

[2] Cai D., Qiu J., Cao Z., McAtee M., Bregman B. S., Filbin M. T. (2001). Neuronal cyclic AMP controls the developmental loss in ability of axons to regenerate. *Journal of Neuroscience*.

[3] Pearse D. D., Sanchez A. R., Pereira F. C. (2007). Transplantation of Schwann cells and/or olfactory ensheathing glia into the contused spinal cord: Survival, migration, axon association, and functional recovery. *Glia*.

[4] Pearse D. D., Pereira F. C., Marcillo A. E. (2004). cAMP and Schwann cells promote axonal growth and functional recovery after spinal cord injury. *Nature Medicine*.

[5] Gao Y., Deng K., Hou J. (2004). Activated CREB is sufficient to overcome inhibitors in myelin and promote spinal axon regeneration in vivo. *Neuron*.

[6] Cai D., Deng K., Mellado W., Lee J., Ratan R. R., Filbin M. T. (2002). Arginase I and polyamines act downstream from cyclic AMP in overcoming inhibition of axonal growth MAG and myelin in vitro. *Neuron*.

[7] Degeorge M. L., Marlowe D., Werner E. (2011). Combining glial cell line-derived neurotrophic factor gene delivery (AdGDNF) with L-arginine decreases contusion size but not behavioral deficits after traumatic brain injury. *Brain Research*.

[8] Kuo H.-S., Tsai M.-J., Huang M.-C. (2011). Acid fibroblast growth factor and peripheral nerve grafts regulate Th2 cytokine expression, macrophage activation, polyamine synthesis, and neurotrophin expression in transected rat spinal cords. *Journal of Neuroscience*.

[9] Kuo H.-S., Tsai M. J., Huang M.-C. (2007). The combination of peripheral nerve grafts and acidic fibroblast growth factor enhances arginase I and polyamine spermine expression in transected rat spinal cords. *Biochemical and Biophysical Research Communications*.

[10] Park Y. M., Han S. H., Seo S. K., Park K. A., Lee W. T., Lee J. E. (2015). Restorative benefits of transplanting human mesenchymal stromal cells overexpressing arginine decarboxylase genes after spinal cord injury. *Cytotherapy*.

[11] Morrissey T. K., Kleitman N., Bunge R. P. (1991). Isolation and functional characterization of Schwann cells derived from adult peripheral nerve. *Journal of Neuroscience*.

[12] Meijs M. F. L., Timmers L., Pearse D. D. (2004). Basic fibroblast growth factor promotes neuronal survival but not behavioral recovery in the transected and Schwann cell implanted rat thoracic spinal cord. *Journal of Neurotrauma*.

[13] Takami T., Oudega M., Bates M. L., Wood P. M., Kleitman N., Bunge M. B. (2002). Schwann cell but not olfactory ensheathing glia transplants improve hindlimb locomotor performance in the moderately contused adult rat thoracic spinal cord. *Journal of Neuroscience*.

[14] Gruner J. A. (1992). A monitored contusion model of spinal cord injury in the rat. *Journal of Neurotrauma*.

[15] Barakat D. J., Gaglani S. M., Neravetla S. R. (2005). Survival, integration, and axon growth support of glia transplanted into the chronically contused spinal cord. *Cell Transplantation*.

[16] Lo T. P., Cho K.-S., Garg M. S. (2009). Systemic hypothermia improves histological and functional outcome after cervical spinal cord contusion in rats. *Journal of Comparative Neurology*.

[17] Kudou M., Shiraki K., Fujiwara S., Imanaka T., Takagi M. (2003). Prevention of thermal inactivation and aggregation of lysozyme by polyamines. *European Journal of Biochemistry*.

[18] Pearse D. D., Lo T. P., Cho K. S. (2005). Histopathological and behavioral characterization of a novel cervical spinal cord displacement contusion injury in the rat. *Journal of Neurotrauma*.

[19] Kanno H., Pressman Y., Moody A. (2014). Combination of engineered Schwann cell grafts to secrete neurotrophin and chondroitinase promotes axonal regeneration and locomotion after spinal cord injury. *Journal of Neuroscience*.

[20] Basso D. M., Beattie M. S., Bresnahan J. C. (1995). A sensitive and reliable locomotor rating scale for open field testing in rats. *Journal of Neurotrauma*.

[21] Schaal S. M., Kitay B. M., Cho K. S. (2007). Schwann cell transplantation improves reticulospinal axon growth and forelimb strength after severe cervical spinal cord contusion. *Cell Transplantation*.

[22] Flora G., Joseph G., Patel S. (2013). Combining neurotrophin-transduced Schwann cells and rolipram to promote functional recovery from subacute spinal cord injury. *Cell Transplantation*.

[23] Wood P. L., Khan M. A., Kulow S. R., Mahmood S. A., Moskal J. R. (2006). Neurotoxicity of reactive aldehydes: the concept of ‘aldehyde load’ as demonstrated by neuroprotection with hydroxylamines. *Brain Research*.

[24] Deng K., He H., Qiu J., Lorber B., Bryson J. B., Filbin M. T. (2009). Increased synthesis of spermidine as a result of upregulation of arginase I promotes axonal regeneration in culture and in vivo. *The Journal of Neuroscience*.

[25] Bellé N. A. V., Dalmolin G. D., Fonini G., Rubin M. A., Rocha J. B. T. (2004). Polyamines reduces lipid peroxidation induced by different pro-oxidant agents. *Brain Research*.

[26] Imagama T., Ogino K., Takemoto K. (2012). Regulation of nitric oxide generation by up-regulated arginase I in rat spinal cord injury. *Journal of Clinical Biochemistry and Nutrition*.

[27] Huang Y., Higginson D. S., Hester L., Myung H. P., Snyder S. H. (2007). Neuronal growth and survival mediated by eIF5A, a polyamine-modified translation initiation factor. *Proceedings of the National Academy of Sciences of the United States of America*.

[28] Buss A., Pech K., Kakulas B. A. (2007). Growth-modulating molecules are associated with invading Schwann cells and not astrocytes in human traumatic spinal cord injury. *Brain*.

[29] Oudega M. (2007). Schwann cell and olfactory ensheathing cell implantation for repair of the contused spinal cord. *Acta Physiologica*.

[30] Banan A., McCormack S. A., Johnson L. R. (1998). Polyamines are required for microtubule formation during gastric mucosal healing. *The American Journal of Physiology—Gastrointestinal and Liver Physiology*.

[31] Kamińska B., Kaczmarek L., Grzelakowska-Sztabert B. (1992). Inhibitors of polyamine biosynthesis affect the expression of genes encoding cytoskeletal proteins. *FEBS Letters*.

[32] McCormack S. A., Ray R. M., Blanner P. M., Johnson L. R. (1999). Polyamine depletion alters the relationship of F-actin, G-actin, and thymosin beta4 in migrating IEC-6 cells. *American Journal of Physiology*.

[33] Hirata H., Hibasami H., Hineno T. (1995). Role of ornithine decarboxylase in proliferation of Schwann cells during Wallerian degeneration and its enhancement by nerve expansion. *Muscle and Nerve*.

[34] Ohkaya S., Hibasami H., Hirata H. (1997). Nerve expansion in nerve regeneration: effect of time on induction of ornithine decarboxylase and Schwann cell proliferation. *Muscle and Nerve*.

[35] Thomas T., Thomas T. J. (2001). Polyamines in cell growth and cell death: molecular mechanisms and therapeutic applications. *Cellular and Molecular Life Sciences*.

[36] Nishimura K., Murozumi K., Shirahata A., Park M. H., Kashiwagi K., Igarashi K. (2005). Independent roles of eIF5A and polyamines in cell proliferation. *Biochemical Journal*.

[37] Parkkinen J. J., Lammi M. J., Ågren U. (1997). Polyamine-dependent alterations in the structure of microfilaments, Golgi apparatus, endoplasmic reticulum, and proteoglycan synthesis in BHK cells. *Journal of Cellular Biochemistry*.

[38] Lopatin A. N., Makhina E. N., Nichols C. G. (1994). Potassium channel block by cytoplasmic polyamines as the mechanism of intrinsic rectification. *Nature*.

[39] Komulainen H., Bondy S. C. (1987). Transient elevation of intrasynaptosomal free calcium by putrescine. *Brain Research*.

[40] Khan N. A., Moulinoux J. P., Deschaux P. (1996). Putrescine modulation of depolarization-induced [^3^H]serotonin release from fish brain synaptosomes. *Neuroscience Letters*.

[41] Ribotta M. G., Provencher J., Feraboli-Lohnherr D., Rossignol S., Privát A., Orsal D. (2000). Activation of locomotion in adult chronic spinal rats is achieved by transplantation of embryonic raphe cells reinnervating a precise lumbar level. *The Journal of Neuroscience*.

[42] Eaton M. J., Pearse D. D., McBroom J. S., Berrocal Y. A. (2008). The combination of human neuronal serotonergic cell implants and environmental enrichment after contusive SCI improves motor recovery over each individual strategy. *Behavioural Brain Research*.

[43] Schorscher-Petcu A., Austin J.-S., Mogil J. S., Quirion R. (2009). Role of central calcitonin gene-related peptide (CGRP) in locomotor and anxiety- and depression-like behaviors in two mouse strains exhibiting a CGRP-dependent difference in thermal pain sensitivity. *Journal of Molecular Neuroscience*.

[44] Krenz N. R., Weaver L. C. (1998). Sprouting of primary afferent fibers after spinal cord transection in the rat. *Neuroscience*.

